# HIN8/453: State of Global Outbreak Reporting on the Internet

**DOI:** 10.2196/jmir.1.suppl1.e39

**Published:** 1999-09-19

**Authors:** J Woodall

**Affiliations:** ^1^Federal University of Rio de JaneiroRio de JaneiroBrazil

**Keywords:** Outbreak, Emerging Infectious Disease, Internet

## Abstract

The amount of information on current outbreaks of emerging infectious diseases posted on the Internet in time to be useful for prevention and control, is slowly improving. Notable examples have been the official Web site of the health department of Hong Kong regarding the ´chicken flu´ influenza A (H5N1) outbreak of 1997-98 and Malaysia´s on the viral encephalitis outbreak of 1998-99. WHO has improved its timeliness with input from the Canadian initiative GPHIN (Global Public Health Intelligence Network), and other national and international Web sites have increased their reporting. However, ProMED-mail remains the only freely and publicly available independent Internet service giving early warning of new outbreaks of human diseases world-wide which may be emerging threats. It also reports outbreaks in livestock, wildlife and food crops that may menace human health. A similar list in Spanish and Portuguese, ProMED-PORT, is in operation, also Web sites with translations of outbreak reports in Chinese and Japanese, but regions and countries have been slow to copy this demonstrably effective early warning model. The current situation is reviewed.

